# The pseudogene DUXAP10 contributes to gefitinib resistance in NSCLC by repressing OAS2 expression: DUXAP10 contributes to gefitinib resistance by repressing OAS2

**DOI:** 10.3724/abbs.2022176

**Published:** 2022-11-25

**Authors:** Shengnan Ren, Ya Zhu, Siying Wang, Qinqiu Zhang, Niu Zhang, Xiaoteng Zou, Chenchen Wei, Zhaoxia Wang

**Affiliations:** 1 Cancer Medical Center the Second Affiliated Hospital of Nanjing Medical University Nanjing 210011 China; 2 Department of Oncology Sir Run Run Hospital Nanjing Medical University Nanjing 210011 China

**Keywords:** NSCLC, gefitinib resistance, DUXAP10, EZH2, OAS2

## Abstract

Gefitinib, an epidermal growth factor receptor-tyrosine kinase inhibitor (EGFR-TKI),is the currently recommended first-line therapy for advanced EGFR-mutant lung cancer, and understanding the mechanism of resistance is the key to formulating therapeutic strategies for EGFR-TKIs. In this study, we evaluate the expression patterns and potential biological functions of the pseudogene DUXAP10 in gefitinib resistance. We find that pseudogene DUXAP10 expression is significantly upregulated in NSCLC gefitinib-resistant cells and tissues. Gain and loss of function assays reveal that knockdown of DUXAP10 by siRNA reverses gefitinib resistance both *in vitro* and *in vivo*. Furthermore, DUXAP10 interacts with the histone methyltransferase enhancer of zeste homolog 2 (EZH2) to repress the expression of 2′,5′-oligoadenylate synthetase (OAS2). Overall, our study highlights the pivotal role of DUXAP10 in gefitinib resistance, and the DUXAP10/EZH2/OAS2 axis might be a promising therapeutic target to overcome acquired gefitinib resistance in NSCLC.

## Introduction

Lung cancer is the leading cause of cancer-related mortality worldwide. In 2021, the number of lung cancer death was predicted to be 131,880 in the United States [1]. Notably, most patients are diagnosed at an advanced stage, and the overall survival treated with platinum-based chemotherapy is less than 2 years [ 2, 3] . With the development of next-generation sequencing (NGS), we have transformed our view from histopathological classification to precise molecular and genetic subsets. Target-specific small-molecule inhibitors, such as the epidermal growth factor receptor (EGFR) inhibitor gefitinib, have been demonstrated to improve the response rate and survival rate compared with chemotherapy [4], which has changed the treatment strategy for EGFR mutation-positive advanced NSCLC patients [ 5, 6] . Unfortunately, all patients inevitably develop acquired resistance to gefitinib after 9 to 14 months of treatment [7]. Although third-generation EGFR-TKIs can overcome the T790M mutation, almost 50% of resistance mechanisms are still unclear. Therefore, it is urgent to deepen our understanding of gefitinib resistance and identify novel therapeutic targets.

Pseudogene was first introduced by Jacqu in 1977. There is a 16-bp deletion and 14-bp mismatch at its 5 ′end, which makes it lose coding ability and serve as a homologous gene copy as its counterpart genome [8]. Pseudogenes were initially regarded as nonfunctional relics littering that did not have protein-coding abilities on account of several endogenous inactivating gene mutations [9]. Recently, an increasing number of studies have revealed that pseudogenes are involved in regulating transcription and posttranscriptional levels through parental-gene-dependent or parental-gene-independent mechanisms [10]. In cancer, pseudogenes play an important role in tumorigenesis, development and prognosis evaluation. For example, Yndestad *et al*. [11] reported that pseudogene PTENP1 upregulation increased PTEN transcript levels and inhibited tumor progression in ER-negative breast cancers. HMGA1P6, which is transcriptionally activated by the oncogene MYC, promotes ovarian cancer cell malignancy by acting as a ceRNA that enhances HMGA1/2 expression [12]. Moreover, the pseudogene PDIA3P1 acts as an oncogene to promote proliferation and inhibit apoptosis of hepatocellular carcinoma by inhibiting the p53 pathway [13]. Recently, the pseudogene DUXAP10 was shown to be overexpressed in various human cancers and emerged as a key cancer regulator [ 14– 16] . The pseudogene DUXAP10 is located on human chromosome 14q11.2 with a total length of 2398 bp, which is significantly upregulated in human cancers and might be a risk factor for poor prognosis [17]. However, the expression patterns and potential biologic functions of DUXAP10 in gefitinib resistance are still unknown.

In the present study, we identified that DUXAP10 contributes to gefitinib resistance by regulating 2′,5′-oligoadenylate synthetase (OAS2). DUXAP10 may be a novel predictive biomarker for gefitinib resistance and a promising therapeutic target for reversing gefitinib resistance.

## Materials and Methods

### Tissue samples

A cohort of 25 advanced NSCLC patients from the Second Affiliated Hospital of Nanjing Medical University in 2015–2017 who had EGFR-sensitive mutations (exon 19 deletion or exon 21 L858R mutation) was included in this study. None of these patients had received surgery or chemoradiotherapy. The expressions of DUXAP10 and OAS2 were detected by quantitative real-time PCR (qRT-PCR) before gefitinib treatment and after the development of acquired resistance to gefitinib. The tissue samples were obtained by tracheoscopy, percutaneous lung puncture and operation, stored in liquid nitrogen and then transferred to a –80°C cryocooler. This study was approved by the Research Ethics Committee of the Second Affiliated Hospital of Nanjing Medical University. The patients/participants provided written informed consent to participate in this study.

### Cell culture

The human NSCLC PC9 cell line was purchased from the Cell Bank of the Chinese Academy of Sciences (Shanghai, China). The gefitinib-resistant PC9/GR cell line was established by the stepwise escalation method: parental PC9 cells were cultured with stepwise escalation of the concentration of gefitinib from 5 nM to 5 μM over 6 months. PC9 and PC9/GR cells were cultured in DMEM (Gibco, Grand Island, USA) supplemented with 10% fetal bovine serum and antibiotics (100 IU/mL penicillin and 100 μg/mL streptomycin; Gibco, Grand Island, USA) and maintained in a humidified incubator with 5% CO _2_ at 37°C.

### RNA isolation and qRT-PCR

Total RNA was extracted from tissues or cultured cells using TRIzol reagent (Invitrogen, Carlsbad, USA). The isolated RNA (1 μg) was reversely transcribed to cDNA by using random primers under standard conditions with the PrimeScript RT Reagent Kit (Takara, Dalian, China). qRT-PCR analyses were performed using SYBR Green (Takara) following the manufacturer’s protocol. Real-time PCR was performed in triplicate on the Applied Biosystems 7300 Real-Time PCR System (Applied Biosystems, Foster City, USA) with the following condition: pre-denaturation at 95°C for 2 min; 95°C for 5s, 60°C for 34 s, 68°C for 45 s, 40 cycles. The results were normalized to the expression of *GAPDH* or *U6* snRNA, and data were calculated based on 2 ^−ΔΔCT^ method. The primer sequences are listed in Table 1.

**Table:** **Table 1** Sequences of primers used in this study

Name	Sequence (5′→3′)
For qPCR	
DUXAP 10 F	CTGTAGGAGGCCAAGACAGG
DUXAP10 R	CATTGTCTCAAGGTCTGCTGAA
GAPDH F	AGAAGGCTGGGGCTCATTTG
GAPDH R	AGGGGCCATCCACAGTCTTC
U 6 F	CTCGCTTCGGCAGCACACTGC
U6 R	AACGCTTCACGAATTTGCGT
DUSP 1 F	AGTACCCCACTCTACGATCAGG
DUSP1 R	GAAGCGTGATACGCACTGC
ICAM F	ATGCCCAGACATCTGTGTCC
ICAM R	GGGGTCTCTATGCCCAACAA
SESN 2 F	AAGGACTACCTGCGGTTCG
SESN2 R	CGCCCAGAGGACATCAGTG
ZNF 367 F	GGCTTCAGCGACTTCATGGT
ZNF367 R	GTCGGATTCCATCCTTGAGGT
OAS 2 F	CCAATCAGCGAGGCCAGTAAT
OAS2 R	GCAGAACATTCCAAGATGGT
SATB 2 F	GCAGTTGGACGGCTCTCTT
SATB2 R	CACCTTCCCAGCTTGATTATTCC
ANKRD 1 F	AGTAGAGGAACTGGTCACTGG
ANKRD1 R	TGTTTCTCGCTTTTCCACTGTT
LATS 2 F	ACCCCAAAGTTGGACCTTA
LATS2 R	CATTTGCCGGTTCACTTCTGC
EZH 2 F	AATCAGAGTACATGCGACTGAGA
EZH2 R	GCTGTATCCTTCGCTGTTTCC
For CHIP qPCR	
OAS 2 F1	GCTGCCATTTTCCCCCAGCT
OAS2 R1	CCCATTGTGAGGGCTTCATAC
OAS 2 F2	TCTCTGCCGCTTTCCCTGAAT
OAS2 R2	TCGTAGGATTAGCTTGATGAT
OAS 2 F3	TGTTTTCTTGCCCTTGCGAGG
OAS2 R3	TCAGGTTTTGGTCTGCTTCTG
OAS 2 F4	GGGAAGAGCATTTGAGCTTA
OAS2 R4	CTGCAGCGAGCTTACCAACT

### Plasmid constructs and cell transfection

The DUXAP10 and OAS2 cDNAs were synthesized and ligated into the expression vector pcDNA3.1 (Generay Biotech, Shanghai,China). Plasmids (pcDNA3.1-DUXAP10, pcDNA3.1-OAS2, and empty vector) were transfected into PC9 and PC9/GR cells using X-treme GENE HP DNA transfection reagent (Roche, Basel, Switzerland). Three DUXAP10 siRNAs (si-DUXAP10 #1, #2, and #3) and one scrambled siRNA purchased from Invitrogen were transfected into PC9/GR cells using Lipofectamine 2000 (Invitrogen, Carlsbad, USA). The target sequences for siRNAs are listed in Table 2. After transfection for 48 h, the cells were harvested for qRT-PCR and other subsequent experiments.

**Table:** **Table 2** Sequences of siRNAs used in this study

Name	Sequence (5′→3′)
si-DUXAP10 #1	GGAACUUCCCAAACCUCCAUGAUUU
	AAAUCAUGGAGGUUUGGGAAGUUCC
si-DUXAP10 #2	CAGCAUACUUCAAAUUCACAGCAAA
	UUUGCUGUGAAUUUGAAGUAUGCUG
si-DUXAP10 #3	AGUUGUUUGUUAGAAUACUGGUGCU
	AGCACCAGUAUUCUAACAAACAACU
si-EZH2 #1	GAGGUUCAGACGAGCUGAUUU
	AUCAGCUCGUCUGAACCUCTT
si-EZH2 #2	GACTCTGAATGCAGTTGCT
	GCAAATTCTCGGTGTCAAA
si-NC	UUCUCCGAACGUGUCACGUTT
	ACGUGACACGUUCGGAGAATT

### Cell proliferation assay

Cell proliferation was tested using the Cell Counting Kit-8 (CCK8; APExBIO, Houston, USA). PC9 and PC9/GR cells transfected with empty vector, pcDNA3.1-DUXAP10, si-NC, or si-DUXAP10 were plated in 96-well plates. DMEM containing different concentrations of gefitinib was added and incubated for 72 h after the cells adhered to the wells. Then, CCK8 reagent was added to each well and incubated for 2 h. The optical density (OD) was measured with a microplate reader (Bio-Rad, Hercules, USA) at 450 nm. In the colony formation assay, cells were collected at 24 h after transfection, seeded into 6-well plates, maintained in medium containing 10% FBS and exposed to gefitinib for 48 h. Then, the drugs were washed away, and the medium was refreshed every 4 days. Then, the colonies were fixed with methanol and stained with 0.1% crystal violet (Sigma-Aldrich, St Louis, USA) for 15 min. Finally, the numbers of colonies were counted with an inverted microscope (Nikon, Tokyo, Japan).

### Ethynyl deoxyuridine (EdU) (red)/DAPI (blue) immunostaining assay

The specific reaction of fluorescent dye with EdU can directly and accurately detect DNA replication activity. EdU assay was performed using the Click-iT ^®^ EdU imaging kit (Invitrogen, Carlsbad, USA) according to the manufacturer’s protocol. Briefly, PC9/GR cells were cultured at 5×10 ^4^ cells/well in 24-well plates and transfected with siRNA. Two hundred microliters of EdU culture medium was added to each well and incubated for 2 h at 37°C under 5% CO _2_. Next, the cultured cells were fixed with 4% paraformaldehyde for 30 min at room temperature and treated with 0.5% Triton X-100. The samples were stained with Apollo reaction solution at room temperature for 30 min in the dark. DAPI was added to each well to label the cell nuclei. The number of EdU-positive cells was counted under a fluorescence microscope (CKX41-F32FL; Olympus, Tokyo, Japan). Five fields of view were randomly assessed for each treatment group, and the percentage of EdU-positive cells was calculated.

### Flow cytometric analysis

PC9 and PC9/GR cells transfected with pcDNA-DUXAP10 and si-DUXAP10 were treated with gefitinib for 48 h. Then, the cells were harvested by trypsinization and double-stained with fluorescein isothiocyanate (FITC)-Annexin V and propidium iodide using the FITC Annexin V apoptosis detection kit (BD Biosciences, Franklin Lakes, USA). Cells were analysed by flow cytometry with a FACScan ^®^ flow cytometer (BD Biosciences) equipped with CellQuest software (BD Biosciences). The proportions of apoptotic cells were calculated and compared with control transfectants. For cell cycle analysis, cells were stained with PI using the CycleTEST ^TM^ Plus DNA Reagent Kit (BD Biosciences) following the manufacturer’s protocol, and treated cells were analysed by FACScan ^®^ flow cytometry. The relative proportions of cells in G0/G1, S, and G2/M phases were estimated and compared.

### Tumor formation assay *in vivo*

Male athymic BALB/c mice (5 weeks old; Animal Core Facility of Nanjing Medical University) were maintained under specific pathogen-free conditions and manipulated following protocols approved by the Shanghai Medical Experimental Animal Care Commission. PC9/GR cells were stably transfected with sh-DUXAP10 and empty vector, harvested from 6-well cell culture plates, washed with phosphate-buffered saline, and resuspended at a concentration of 1×10 ^8^ cells/mL. A volume of 100 μL of suspended cells was subcutaneously injected into either side of the posterior flank of each mouse. Five days after tumor cell inoculation, gefitinib treatment was administered by oral gavage every day at a dose of 10 mg/kg. Tumor growth was examined every 3 days, and tumor volumes were calculated using the equation *V*=0.5×length×width ^2^. At 18 days postinjection, the mice were euthanized, and the tumors were resected from all mice for immunohistochemical (IHC) staining.

### Transcriptome sequencing

Total RNA from PC9/GR cells with DUXAP10 knockdown or control cells was isolated and quantified. The concentration of each sample was measured with NanoDrop 2000 (Thermo Scientific, Waltham, USA), and the quality was assessed with Agilent 2200 (Agilent, Santa Clara, USA). The sequencing library of each RNA sample was established using the Ion Proton Total RNA-Seq Kit v2 (Life Technologies, Carlsbad, USA) according to the protocol provided by the manufacturer. Data are listed in Supplementary Table S1.

### Subcellular fractionation

The separation of nuclear and cytosolic fractions was performed using the PARIS Kit (Life Technologies) following the manufacturer’s protocol. In brief, PC9/GR cells were digested with ACCUTASE, and then lysed with the Cell lysis buffer. The cytosolic fraction was collected. The cell lysate was further subject to the Nuclear lysis buffer, and the nuclear fraction was collected through the columns in the kit.

### RIP assay

RIP assay was performed using a Magna RIP RNA-binding protein immunoprecipitation kit (Millipore, Billerica, USA) in accordance with the manufacturer’s instructions. PC9/GR cells at 80%–90% confluency were scraped off the tissue culture plate and lysed in complete RIP lysis buffer. Cell lysate was incubated with RIP buffer containing magnetic beads conjugated with anti-EZH2 (Millipore), anti-LSD1 (Millipore), anti-Ago2 (Cell Signaling Technology), or control IgG (Millipore). Finally, immunoprecipitated RNA was isolated and analysed by quantitative real-time PCR.

### Chromatin immunoprecipitation

PC9/GR cells were treated with formaldehyde with final concentration of 1% for 10 min to generate DNA-protein cross-links. Cells were lysed in SDS Lysis Buffer and sonicated to generate chromatin fragments of 200–300 bp and immunoprecipitated with antibodies specific for EZH2 and H3K27me3 (Millipore) or control IgG (Millipore) as a control. The precipitated chromatin DNA was recovered and analysed by qRT-PCR. The *OAS2* primers amplified the –432 to –570 bp region upstream of the transcription starting site.

### Western blot analysis

PC9 and PC9/GR cells were lysed with RIPA (radioimmunoprecipitation assay) Lysis and Extraction Buffer (Beyotime), supplemented with protease inhibitor cocktail (Roche). Cell protein lysates were separated by 10% SDS-PAGE and then transferred to 0.22 μm polyvinylidene fluoride (PVDF) membranes (Millipore). The membranes were incubated with 5% milk and then with specific antibodies against EZH2 (1:1000; Millipore), OAS2 (1:1000; Cell Signaling Technology, Beverly, USA) or GAPDH (1:1000; Proteintech, Chicago, USA) overnight at 4°C. ECL chromogenic substrate was used to were quantified by densitometry (Quantity One software; Bio-Rad). GAPDH was used as an internal control.

### Statistical analysis

Data are expressed as the mean±SD based on triplicate experiments. One-way ANOVA and Student’s *t*-test were used to analyse the comparison among the groups. All statistical analyses were conducted by using SPSS 18.0 software (IBM; SPSS, Chicago, USA) and GraphPad Prism 7 (GraphPad Software, San Diego, USA). *P*<0.05 was considered statistically significant.

## Results

### PC9/GR is established as a gefitinib-resistant cell line, and DUXAP10 is overexpressed in PC9/GR cells

**Figure 1:**
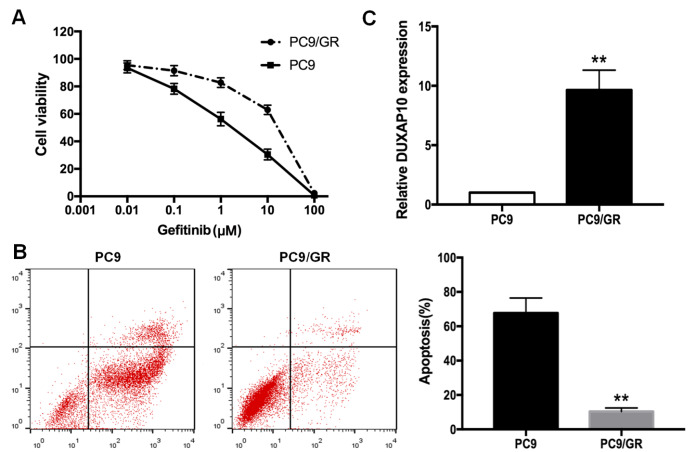
Figure 1 Differences in the resistance of PC9/GR and PC9 cells to gefitinib (A) IC 50 values of gefitinib in PC9/GR and parental PC9 cells measured by CCK8 assay. (B) Flow cytometric analysis of apoptosis in PC9/GR and parental PC9 cells treated with 1 μM gefitinib. (C) Expression of DUXAP10 in PC9 and PC9/GR cells detected by qRT-PCR. ** P<0.01.

To verify the gefitinib resistance of PC9/GR cells, different concentrations of gefitinib (0, 0.01, 0.1, 1, 10, 100 μM) were administered to PC9 and PC9/GR cells for 72 h, and cell viabilities were measured by CCK8 assay to calculate the inhibitory concentration (IC _50_) values of gefitinib in PC9 and PC9/GR cells. As shown in Figure 1A, the IC _50_ value of gefitinib in PC9/GR cells was significantly higher than that in PC9 cells (14.68±1.61 μM vs 1.41±0.41 μM; *P*<0.01). Flow cytometry was performed to evaluate the apoptosis rate of PC9 and PC9/GR cells. As shown in Figure 1B, the apoptosis rate of PC9/GR cells was obviously lower than that of PC9 cells after the initiation of exposure to gefitinib (1 μM) for 48 h ( *P*<0.01). Additionally, we performed genetic sequencing of PC9 and identified a 745–750 deletion in exon 19 ( Supplementary Table S2). qRT-PCR revealed that DUXAP10 was upregulated by 9.63 folds in PC9/GR cells compared with that in PC9 cells ( *P*<0.01, Figure 1C). These data indicated that PC9 cells were sensitive to gefitinib, while PC9/GR cells were resistant to gefitinib, and DUXAP10 is overexpressed in PC9/GR cells.

### DUXAP10 promotes proliferation of lung adenocarcinoma cells

**Figure 2:**
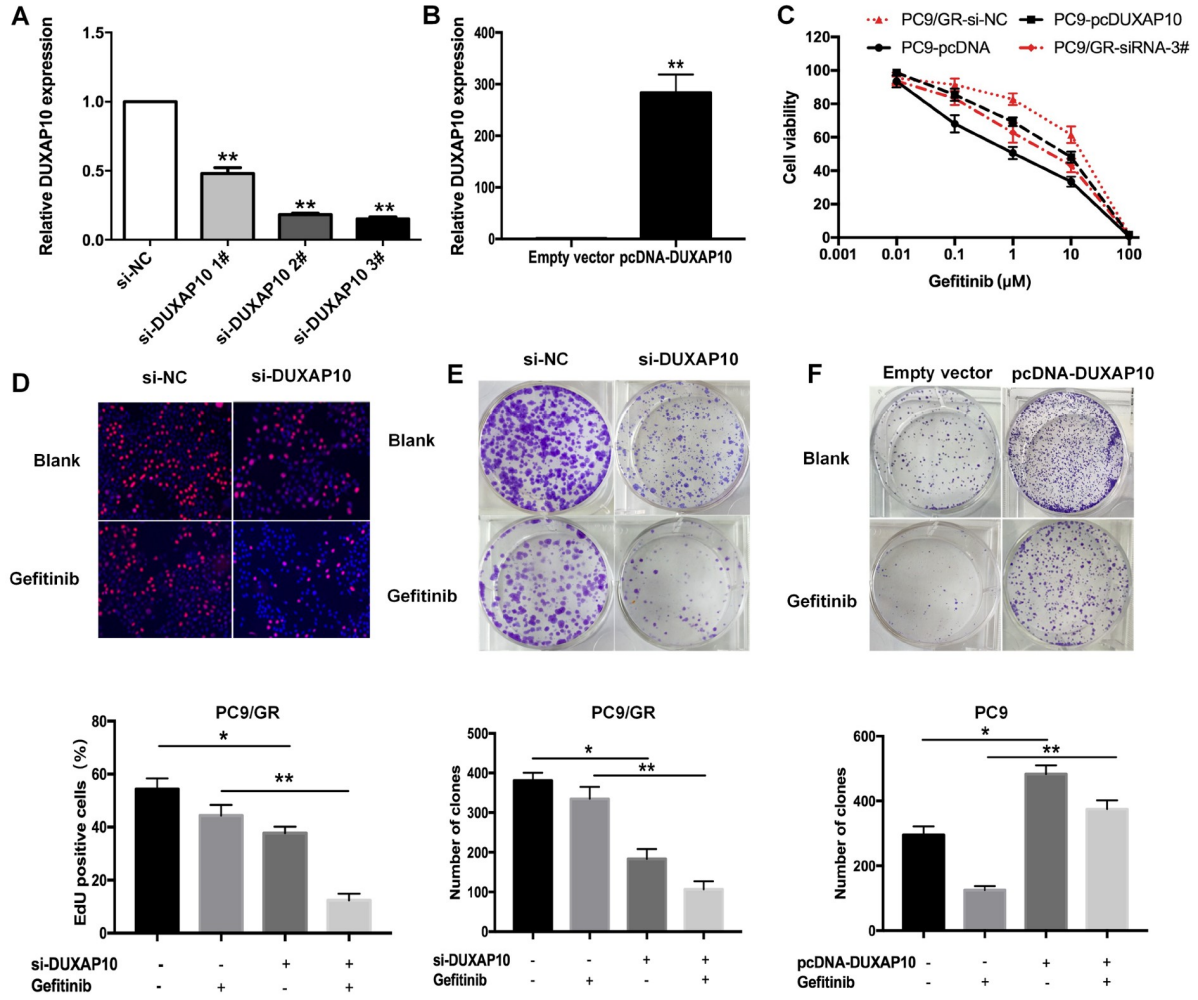
Figure 2 DUXAP10 promotes the proliferation of PC9/GR cells (A) PC9/GR cells were transfected with si-DUXAP10 #1, #2, #3 or scrambled siRNA. (B) PC9 cells were transfected with pcDNA-DUXAP10 or empty vector. (C) IC 50 values of gefitinib in PC9/GR cells with DUXAP10 knockdown and in PC9 cells with DUXAP10 overexpression measured by CCK8 assay. (D,E) EdU and colony formation assays were performed to assess the proliferation of PC9/GR cells transfected with si-DUXAP10 or si-NC in the presence or absence of gefitinib. (F) Colony-forming assay was performed to assess the proliferation of PC9 cells transfected with pcDNA-DUXAP10 or empty vector in the presence or absence of gefitinib. Data are expressed as the mean±SD of three independent experiments. * P<0.05, ** P<0.01.

To explore the effects of DUXAP10 on gefitinib resistance in lung adenocarcinoma cells, we transfected PC9/GR cells with DUXAP10-specific siRNAs to downregulate its expression, and the pcDNA3.1-DUXAP10 vector was transfected into PC9 cells to upregulate its expression. Forty-eight hours after transfection, qRT-PCR showed that the expression of DUXAP10 in PC9/GR cells was significantly downregulated ( Figure 2A), and the expression of DUXAP10 in PC9 cells was increased by 283 folds ( Figure 2B). CCK8 assay indicated that the IC _50_ value of gefitinib in PC9/GR cells transfected with si-DUXAP10 #3 (2.79±0.53 μM) was significantly decreased by 4.21 folds compared to that in PC9/GR cells transfected with si-NC. Correspondingly, the IC _50_ of gefitinib in PC9-pcDUXAP10 cells (4.43±0.50 μM) was increased by approximately 4.72 folds compared with that in PC9-pcDNA cells ( Figure 2C). The immunostaining assay confirmed that the proliferation of PC9/GR cells was inhibited by interference with si-DUXAP10 together with gefitinib treatment ( Figure 2D). To further verify the above results, colony formation assay was performed, and the results indicated that knockdown of *DUXAP10* decreased the colony formation capacity of PC9/GR cells, which was much stronger in the presence of gefitinib ( Figure 2E). These data indicated that PC9 cells overexpressing DUXAP10 was still stronger than that of control cells under the treatment with gefitinib ( Figure 2F).

### Knockdown of *DUXAP10* promotes apoptosis and cell cycle arrest of lung adenocarcinoma cells

**Figure 3:**
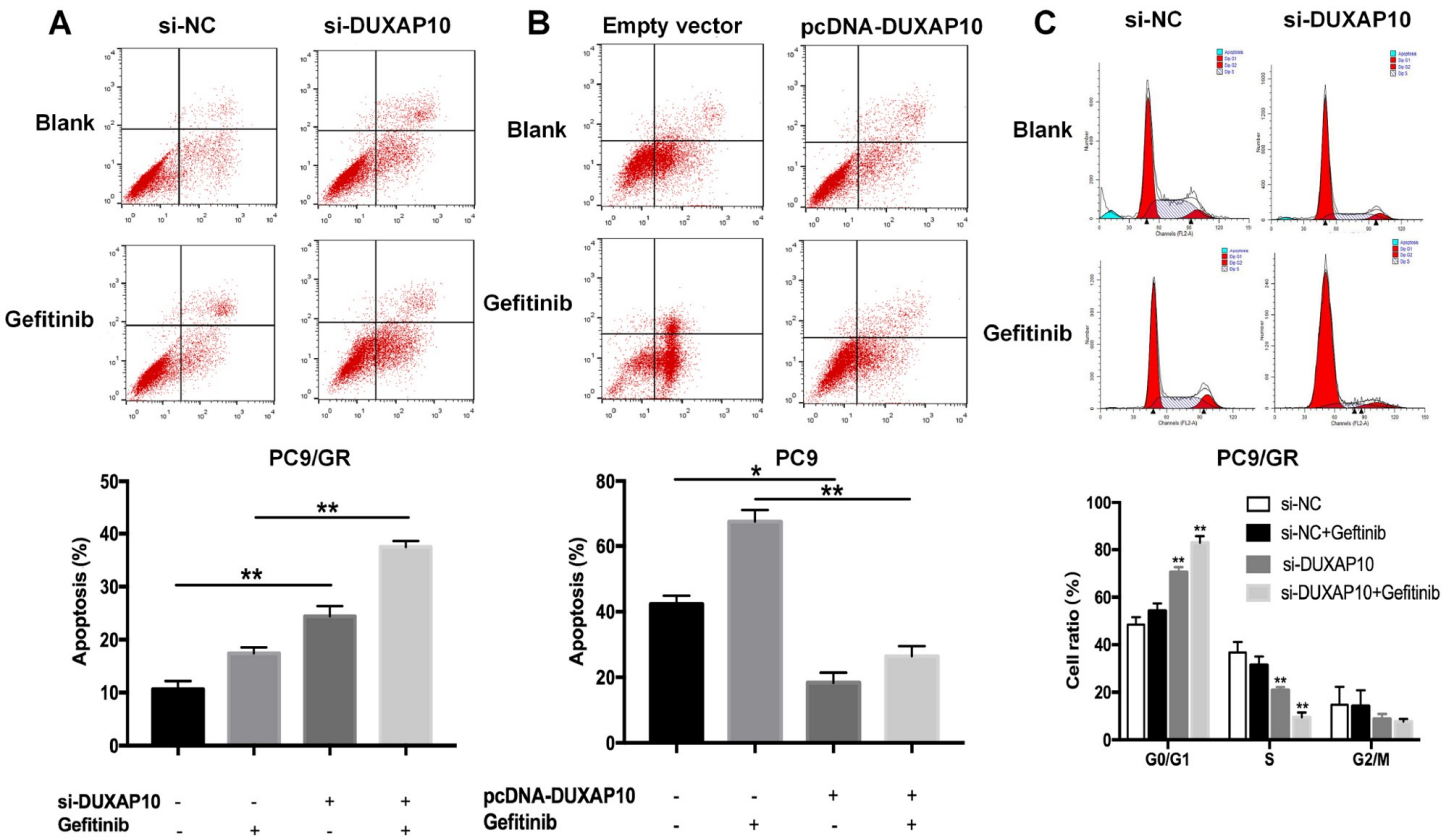
Figure 3 The effects of DUXAP10 on apoptosis and the cell cycle *in vitro* (A) Forty-eight hours after transfection with si-DUXAP10 #3 or scrambled siRNA, the apoptosis rates of PC9/GR cells were detected by flow cytometry in the presence or absence of gefitinib. (B) Cell apoptosis rates of PC9 cells were analysed by flow cytometric analysis. (C) Flow cytometric analysis was used to detect the cell cycle stages of PC9/GR cells. Data are expressed as the mean±SD of three independent experiments. * P<0.05, ** P<0.01.

To analyse the effects of DUXAP10 on apoptosis and cell cycle distribution of lung adenocarcinoma cells treated with gefitinib, flow cytometric analysis was performed. The apoptosis rate was increased after transfection with si-DUXAP10, and the trend was more significant under gefitinib treatment ( Figure 3A). Additionally, the apoptosiss rate of DUXAP10-overexpressing PC9 cells was significantly decreased compared with that of control cells, even after exposure to gefitinib ( Figure 3B). Compared with control cells, knockdown of *DUXAP10* increased the percentage of PC9/GR cells arrested in the G0/G1 phase, and decreased the percentage of cells in the S phase ( Figure 3C). Therefore, DUXAP10 plays an oncogenic role. Overexpression of DUXAP10 reduces the sensitivity of PC9 cells to gefitinib *in vitro*, exhibiting a drug-resistant phenotype. Silencing of *DUXAP10* partially reversed the resistance of PC9/GR cells to gefitinib, exhibiting a drug-sensitive phenotype.

### OAS2 is the key underlying target of DUXAP10

**Figure 4:**
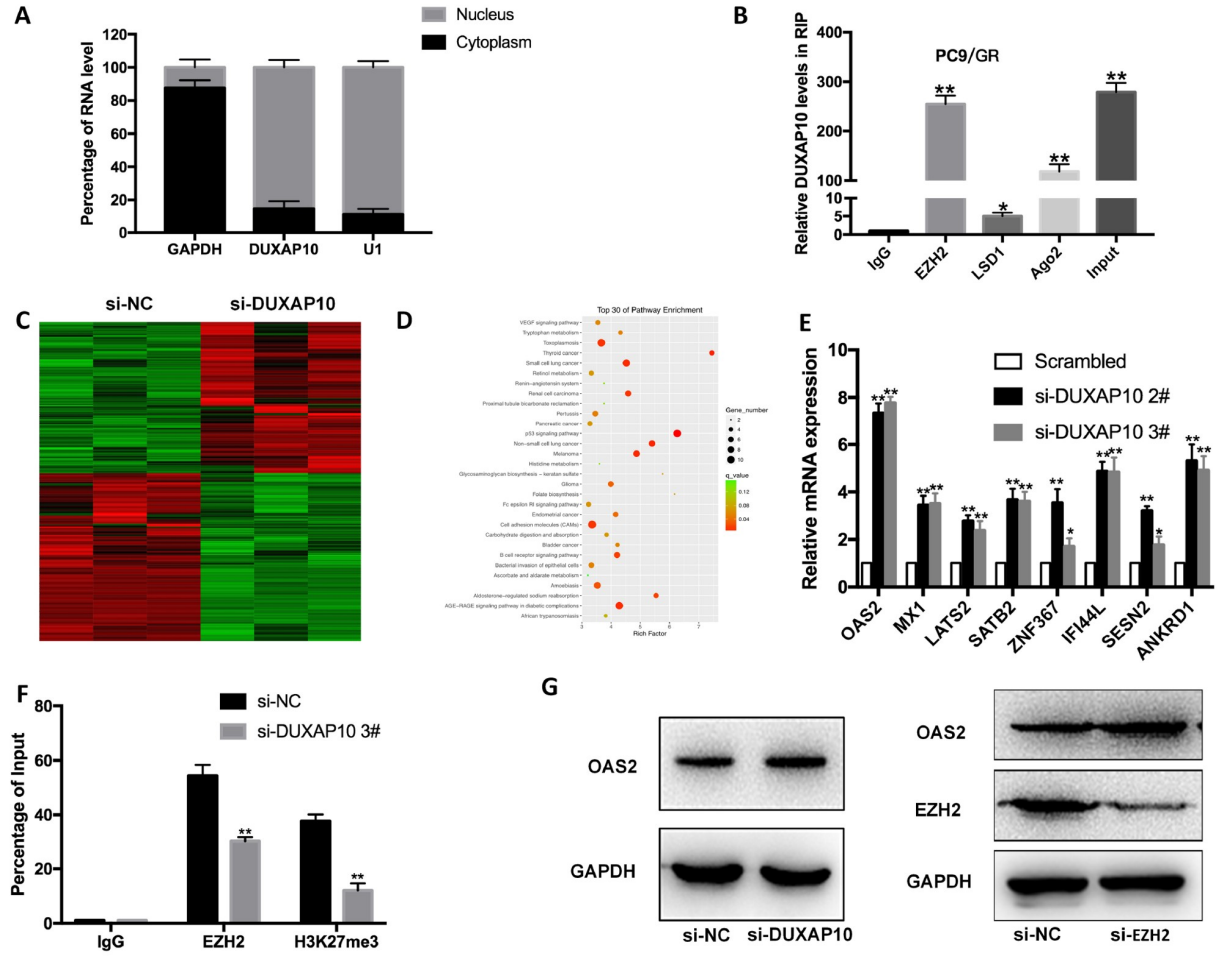
Figure 4 DUXAP10 silences OAS2 transcription by binding to EZH2 (A) qRT-PCR was performed to determine the subcellular localization of DUXAP10 in PC9/GR cells. U1 was used as a nuclear control, and GAPDH was used as a cytoplasmic control. (B) RIP assays were performed in PC9/GR cells to determine DUXAP10 coimmunoprecipitation with EZH2, LSD1 and Ago2. (C) Hierarchically clustered heatmap identified differentially expressed genes in PC9/GR cells and PC9/GR/si-DUXAP10 cells. (D) The top 30 pathways enriched in differentially expressed genes between PC9/GR/siRNA-NC and PC9/GR/si-DUXAP10 cells. (E) qRT-PCR detection of seven representative downstream genes in PC9/GR cells depleted of DUXAP10. (F) ChIP-qRT-PCR of EZH2 occupancy and H3K27me3 binding in the OAS2 promoter in PC9/GR cells transfected with si-DUXAP10 or si-NC; IgG was used as a negative control. (G) Western blot analysis of EZH2 and OAS2 protein levels in PC9/GR cells transfected with si-EZH2. * P<0.05, ** P<0.01.

To explore the molecular mechanism of DUXAP10 in gefitinib-resistant PC9/GR cells, we carried out a nuclear cytoplasmic separation experiment to detect the subcellular localization of DUXAP10 in cells. DUXAP10 was distributed in both the nucleus and cytoplasm ( Figure 4A), mainly in the nucleus, with an abundance of 89.37%, indicating that DUXAP10 may play a role in regulating gene transcription. PRC2, as the core complex of the Polycomb family, catalyzes the methylation of histone H3 on K27 to silence target genes. EZH2, a core subunit of PRC2, was demonstrated to play an important role in NSCLC drug-resistance progression. To explore whether DUXAP10 regulates target genes by a similar mechanism, RNA immunoprecipitation (RIP) assay was conducted. The results suggested that DUXAP10 could specifically interact with EZH2 and Ago2, and its interaction with EZH2 was more significant ( Figure 4B). To search for the downstream target genes, we transfected PC9/GR cells with si-DUXAP10 and detected the differentially expressed genes between PC9/GR cells transfected with si-DUXAP10 and cells transfected with si-NC. The RNA sequencing results showed that there were 497 differentially expressed transcripts: 236 were upregulated and 261 were downregulated ( Figure 4C and Supplementary Table S2). KEGG enrichment and signal pathway analysis indicated that the differentially expressed genes were mainly related to lung cancer, renal cancer, the p53 pathway and cell adhesion molecules ( Figure 4D). qRT-PCR was used to screen seven tumor suppressor genes in PC9/GR cells, among which *OAS2* was upregulated most in DUXAP10-depleted PC9/GR cells ( Figure 4E). Furthermore, ChIP assay confirmed that DUXAP10 could recruit EZH2 to the promoter region of *OAS2*, thereby inhibiting OAS2 transcription ( Figure 4F). Western blot analysis subsequently confirmed that after interference with DUXAP10 and EZH2, the expression of OAS2 was increased ( Figure 4G).

### Overexpression of OAS2 reverses PC9/GR cell resistance to gefitinib

**Figure 5:**
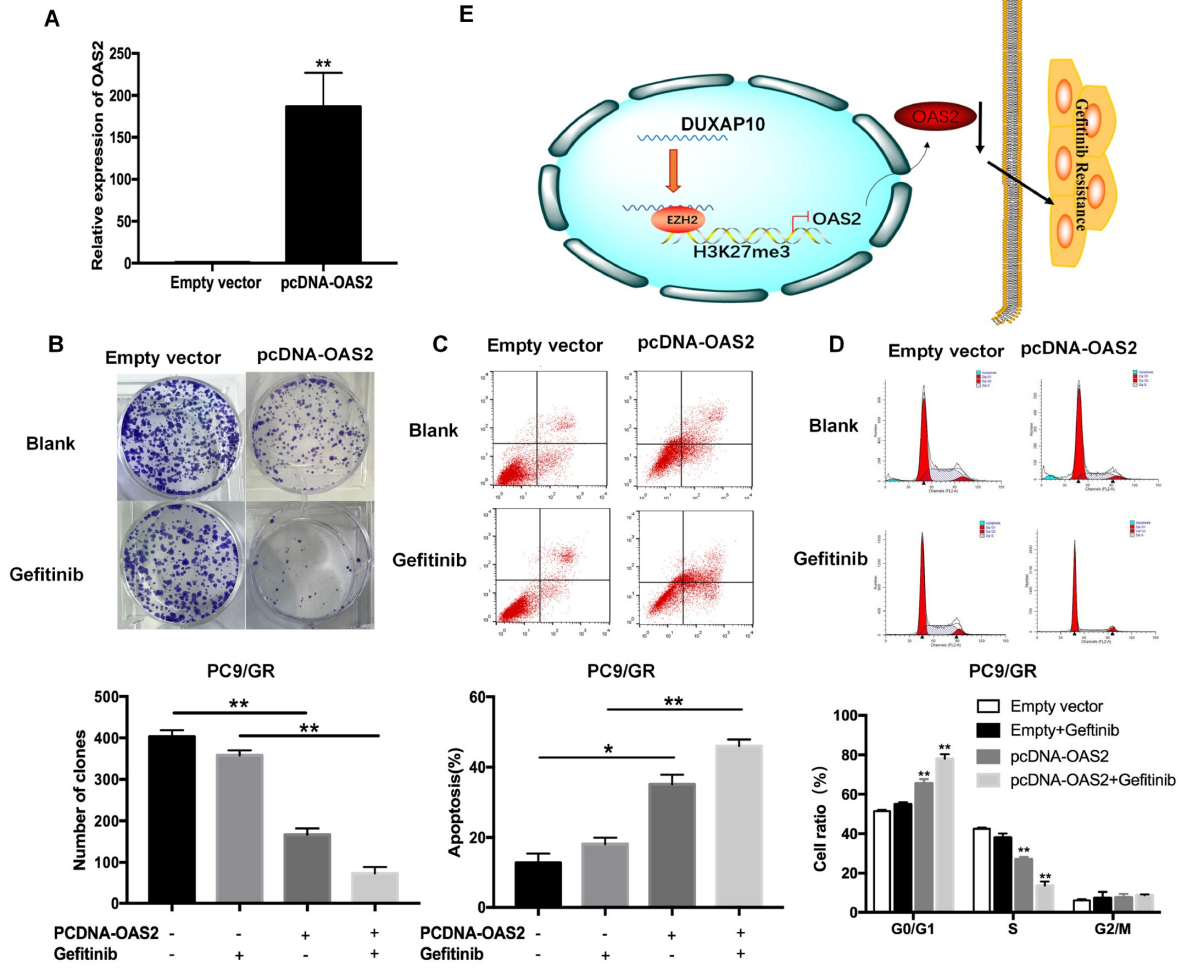
Figure 5 Upregulation of OAS2 reverses the resistance of PC9/GR cells to gefitinib (A) qRT-PCR analysis of OAS2 mRNA in stable PC9/GR cells transfected with empty vector or pcDNA-OAS2. (B) Colony formation assays were used to determine the proliferation of PC9/GR/NC or PC9/GR/OAS2 cells treated with 1 μM gefitinib. (C) Flow cytometric analysis of apoptosis in OAS2-overexpressing cells treated with 1 μM gefitinib. (D) Flow cytometric analysis of the cell cycle in OAS2-overexpressing cells treated with 1 μM gefitinib. (E) Proposed model of DUXAP10’s regulation of gefitinib resistance in NSCLC. * P<0.05, ** P<0.01.

To further investigate the potential role of OAS2 in PC9/GR cells, pcDNA-OAS2 or pcDNA-control was transfected into PC9/GR cells. qRT-PCR results confirmed that the OAS2 expression level in PC9/GR cells was significantly upregulated, up to 180 folds ( Figure 5A). Colony formation assay revealed that OAS2 overexpression inhibited PC9/GR cell proliferation compared with that of control cells with or without gefitinib treatment ( Figure 5B). Moreover, flow cytometry analysis showed that the apoptosis rate of PC9/GR cells ectopically expressing OAS2 was increased, especially in the presence of gefitinib ( Figure 5C), and the cell cycle was arrested in the G0/G1 phase ( Figure 5D). These results indicated that ectopic OAS2 reversed the resistance of PC9/GR cells to gefitinib. The proposed model is shown in Figure 5E.

### DUXAP10 contributes to gefitinib resistance *in vivo*

**Figure 6:**
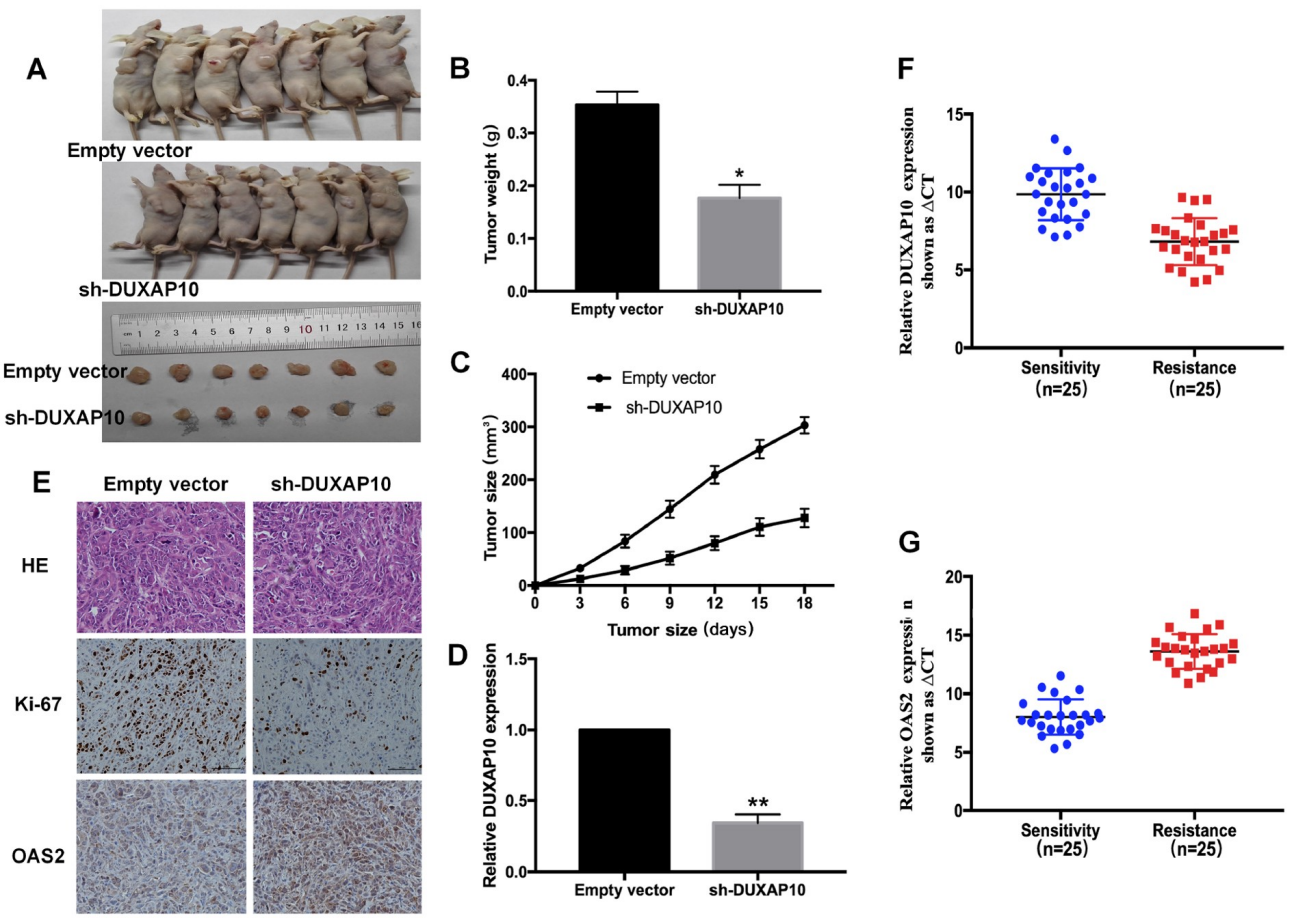
Figure 6 Knockdown of DUXAP10 reverses acquired resistance to gefitinib *in vivo* (A) PC9/GR cells treated with PC9/GR/sh-DUXAP10 or PC9/GR/Empty vector cells were injected into nude mice. After tumorigenesis, the mice were treated with gefitinib (10 mg/kg) by gavage. (B) Tumor weight was measured after inoculation. (C) Tumor volumes were calculated every 3 days. (D) qRT-PCR was used to detect the relative expression of DUXAP10 in the xenograft tumors ( n=7). (E) Immunostaining of OAS2 and Ki-67 expressions in representative tumors derived from PC9/GR/sh-DUXAP10 or PC9/GR/empty vector cells treated with gefitinib. Scale bar: 100 μm. (F) qRT-PCR analysis of the relative expression of DUXAP10 in gefitinib-resistant tissues before and after treatment. (G) OAS2 was detected by qRT-PCR in gefitinib-resistant tissues before and after treatment. * P<0.05, ** P<0.01.

To confirm the biological function of DUXAP10 *in vivo*, we injected PC9/GR cells transfected with sh-DUXAP10 or empty vector into nude mice to establish a subcutaneous tumor model. When the xenograft tumors were successfully inoculated, the mice were treated with gefitinib (10 mg/kg) by gavage ( Figure 6A). As shown in Figure 6B,C, the tumor weight and volume of the sh-DUXAP10 group were lower than those of the empty vector group. qRT-PCR analysis confirmed that the expression of DUXAP10 was significantly downregulated in the excised tumor tissues ( Figure 6D). Immunohistochemistry revealed that sh-DUXAP10-derived tumors expressed a lower level of the proliferation marker Ki-67 and a higher level of OAS2 than the empty vector-transfected tumors ( Figure 6E).

Furthermore, we detected the mRNA expression levels of DUXAP10 and OAS2 in 25 lung adenocarcinoma patients before and after gefitinib treatment. qRT-PCR results showed that the expression of DUXAP10 was significantly increased after the development of gefitinib resistance, but the expression of OAS2 showed the opposite trend ( *P*<0.01, Figure 6F,G). Taken together, these findings suggest that DUXAP10 contributes to gefitinib-resistant tumorigenesis *in vivo*.

## Discussion

Gefitinib, a reversible EGFR-TKI, is the standard first-line therapy for NSCLC in patients harboring sensitizing mutations. Unfortunately, most patients inevitably develop acquired resistance, which presents a crucial challenge in the treatment of NSCLC. The mechanisms of acquired resistance to EGFR-TKIs are particularly complex, including target gene modification, alternative pathway activation and histological transformation [18], which are not yet clearly defined. Our group indicated that long noncoding RNAs play a key role in acquired gefitinib resistance [ 19– 21] . Pseudogenes, as noncoding RNAs, are considered to play a plethora of roles at multiple levels in tumorigenesis and development [22]. In the present study, we established a gefitinib-resistant cell line entitled PC9/GR and found that the pseudogene *DUXAP10* was highly upregulated in gefitinib-resistant cells and tissues. Silencing of *DUXAP10* markedly inhibited PC9/GR cell proliferation, induced cell apoptosis and diminished tumorigenesis *in vivo*. These findings revealed that DUXAP10 may contribute to the acquisition of gefitinib resistance, playing an oncogenic role.

The pseudogene *DUXAP10* is highly expressed in various tumors and functions as an oncogene [ 14, 23] , while the pseudogene-mediated molecular mechanism of acquired resistance to gefitinib has not been reported. In this study, we found that DUXAP10 was mainly distributed in the nucleus and played a biological role at the transcription level. RIP assay showed that DUXAP10 could bind to the core subunit EZH2 of the PRC2 complex. *EZH2* locates on human chromosome 7q35-7q36 and encodes a histone lysine N-methyltransferase which inhibits target gene transcription by trimethylating H3K27 [24]. EZH2 is involved in gefitinib resistance in non-small cell lung cancer. For example, HOTAIR promotes gefitinib resistance through modification of EZH2 and silencing *p16* and *p21* [25]. LINC00665 interacts with EZH2 to regulate the downstream PI3K/Akt pathway, leading to gefitinib resistance [26]. Our findings are consistent with previous studies indicating that EZH2 regulates gefitinib resistance in cancer cells.

We further performed RNA transcriptome sequencing to identify downstream target genes. We selected seven representative tumor suppressor genes for verification. After repeated screening, we identified *OAS2* as a candidate by virtue of the most meaningful upregulation. *OAS2* is an antiviral interferon-stimulated gene that inhibits Zika virus ( *ZIKV*) replication by participating in the expressions of interferon pathway-related genes [27]. In tumors, OAS2 upregulates the expressions of E-cadherin, β-catenin, claudin-1, and snail, induces autophagy, and prolongs recurrence-free survival (RFS) in colorectal cancer [28]. High expression of OAS2 was found to be correlated with the prognosis of breast cancer [29]. These studies demonstrated that OAS2 is a tumor suppressor, and we focused on its role in acquired resistance to gefitinib. ChIP experiments showed that DUXAP10 recruits EZH2 to the promoter region of *OAS2*, trimethylates histone H3K27, and inhibits OAS2 transcription. Then, we explored the biological function of OAS2 in PC9/GR cells. Overexpression of OAS2 induced apoptosis and G0/G1 phase arrest in PC9/GR cells. Tumorigenesis in nude mice showed that OAS2 was increased after DUXAP10 interference, and we further confirmed that OAS2 was significantly decreased in human gefitinib-resistant tissues. Therefore, our findings indicated that OAS2 is involved in gefitinib resistance as a tumor suppressor.

In summary, this study revealed that DUXAP10 contributes to gefitinib resistance by recruiting EZH2 to repress OAS2 expression, forming the DUXAP10-EZH2-OAS2 regulatory axis. Our findings may provide a new tumor therapeutic target for reversing gefitinib resistance.

## Supporting information

087TableS2

087TableS1

## Supplementary Data

Supplementary data is available at *Acta Biochimica et Biphysica Sinica* online.

## References

[1] Siegel RL, Miller KD, Fuchs HE, Jemal A (2021). Cancer statistics, 2021. CA Cancer J Clin.

[2] Schiller JH, Harrington D, Belani CP, Langer C, Sandler A, Krook J, Zhu J (2002). Comparison of four chemotherapy regimens for advanced non–small-cell lung cancer. N Engl J Med.

[3] Ohe Y, Ohashi Y, Kubota K, Tamura T, Nakagawa K, Negoro S, Nishiwaki Y (2007). Randomized phase III study of cisplatin plus irinotecan versus carboplatin plus paclitaxel, cisplatin plus gemcitabine, and cisplatin plus vinorelbine for advanced non-small-cell lung cancer: Four-Arm Cooperative Study in Japan. Ann Oncol.

[4] Maemondo M, Inoue A, Kobayashi K, Sugawara S, Oizumi S, Isobe H, Gemma A (2010). Gefitinib or chemotherapy for non–small-cell lung cancer with mutated EGFR. N Engl J Med.

[5] Herbst RS, Heymach JV, Lippman SM (2008). Lung cancer. N Engl J Med.

[6] Shimada Y, Yanaga K. Molecular diagnosis and targeting for lung cancer. In: Molecular Diagnosis and Targeting for Thoracic and Gastrointestinal Malignancy. Singapore: Springer Singapore 2019, 1–32.

[7] Takeda M, Nakagawa K (2019). First- and second-generation EGFR-TKIs are all replaced to osimertinib in chemo-naive EGFR mutation-positive non-small cell lung cancer?. Int J Mol Sci.

[8] Jacq C, Miller JR, Brownlee GG (1977). A pseudogene structure in 5S DNA of Xenopus laevis. Cell.

[9] Chen X, Wan L, Wang W, Xi WJ, Yang AG, Wang T (2020). Re-recognition of pseudogenes: from molecular to clinical applications. Theranostics.

[10] Shi X, Nie F, Wang Z, Sun M (2016). Pseudogene-expressed RNAs: a new frontier in cancers. Tumor Biol.

[11] Yndestad S, Austreid E, Skaftnesmo KO, Lønning PE, Eikesdal HP (2018). Divergent activity of the pseudogene
*PTENP1* in ER-positive and negative breast cancer. Mol Cancer Res.

[12] Tian X, Song J, Zhang X, Yan M, Wang S, Wang Y, Xu L (2020). MYC-regulated pseudogene HMGA1P6 promotes ovarian cancer malignancy via augmenting the oncogenic HMGA1/2. Cell Death Dis.

[13] Kong Y, Zhang L, Huang Y, He T, Zhang L, Zhao X, Zhou X (2017). Pseudogene PDIA3P1 promotes cell proliferation, migration and invasion, and suppresses apoptosis in hepatocellular carcinoma by regulating the p53 pathway. Cancer Lett.

[14] Lv XY, Ma L, Chen JF, Yu R, Li Y, Yan ZJ, Cheng Y (2017). Knockdown of DUXAP10 inhibits proliferation and promotes apoptosis in bladder cancer cells via PI3K/Akt/mTOR signaling pathway. Int J Oncol.

[15] Xu Y, Yu X, Wei C, Nie F, Huang M, Sun M (2018). Over-expression of oncigenic pesudogene DUXAP10 promotes cell proliferation and invasion by regulating LATS1 and β-catenin in gastric cancer. J Exp Clin Cancer Res.

[16] Wang Z, Ren B, Huang J, Yin R, Jiang F, Zhang Q (2018). LncRNA DUXAP10 modulates cell proliferation in esophageal squamous cell carcinoma through epigenetically silencing p21. Cancer Biol Ther.

[17] Yue C, Ren Y, Ge H, Yan L, Xu Y, Wang G, Wu J (2019). Pseudogene DUXAP10 can be used as a diagnostic and prognostic biomarker in human cancers. J Cell Physiol.

[18] Gao J, Li HR, Jin C, Jiang JH, Ding JY (2019). Strategies to overcome acquired resistance to EGFR TKI in the treatment of non-small cell lung cancer. Clin Transl Oncol.

[19] Xu T, Yan S, Wang M, Jiang L, Ma P, Lu B, Chen Q (2020). LncRNA UCA1 induces acquired resistance to gefitinib by epigenetically silencing CDKN1A expression in non-small-cell lung cancer. Front Oncol.

[20] Chen Z, Chen Q, Cheng Z, Gu J, Feng W, Lei T, Huang J (2020). Long non-coding RNA CASC9 promotes gefitinib resistance in NSCLC by epigenetic repression of DUSP1. Cell Death Dis.

[21] Wang H, Lu B, Ren S, Wu F, Wang X, Yan C, Wang Z (2020). Long noncoding RNA LINC01116 contributes to gefitinib resistance in non-small cell lung cancer through regulating IFI44. Mol Ther Nucleic Acids.

[22] Xiao-Jie L, Ai-Mei G, Li-Juan J, Jiang X (2015). Pseudogene in cancer: real functions and promising signature. J Med Genet.

[23] Lian Y, Xiao C, Yan C, Chen D, Huang Q, Fan Y, Li Z (2018). Knockdown of pseudogene derived from lncRNA DUXAP10 inhibits cell proliferation, migration, invasion, and promotes apoptosis in pancreatic cancer. J Cell Biochem.

[24] Duan R, Du W, Guo W (2020). EZH2: a novel target for cancer treatment. J Hematol Oncol.

[25] Li W, Li Y, Zhang H, Liu M, Gong H, Yuan Y, Shi R (2021). HOTAIR promotes gefitinib resistance through modification of EZH2 and silencing p16 and p21 in non-small cell lung cancer. J Cancer.

[26] Liu X, Lu X, Zhen F, Jin S, Yu T, Zhu Q, Wang W (2019). LINC00665 induces acquired resistance to gefitinib through recruiting EZH2 and activating PI3K/AKT pathway in NSCLC. Mol Ther Nucleic Acids.

[27] Liao X, Xie H, Li S, Ye H, Li S, Ren K, Li Y (2020). 2′, 5′-Oligoadenylate synthetase 2 (OAS2) inhibits zika virus replication through activation of type ι IFN signaling pathway. Viruses.

[28] Kim JC, Ha YJ, Tak KH, Roh SA, Kwon YH, Kim CW, Yoon YS (2018). Opposite functions of GSN and OAS2 on colorectal cancer metastasis, mediating perineural and lymphovascular invasion, respectively. PLoS ONE.

[29] Zhang Y, Yu C (2022). Prognostic characterization of OAS1/OAS2/OAS3/OASL in breast cancer. BMC Cancer.

