# Supplementary material for: The pseudogene DUXAP10 contributes to gefitinib resistance in NSCLC by repressing OAS2 expression: DUXAP10 contributes to gefitinib resistance by repressing OAS2
Source: Acta Biochim Biophys Sin (Shanghai). 2022 Nov 25;55(1):81–90. doi: 10.3724/abbs.2022176 (PMC10157544; doi:10.3724/abbs.2022176)
Supplement: 087TableS1 [file 087TableS1.pdf]

Gene	Gene.ID	AAChange
CYP2B6	CYP2B6:NM 000767.4:exon4	p.Q172H (c.G516T)
CYP2B6	CYP2B6:NM 000767.4:exon5	p.K262R (c.A785G)
CYP2D6	CYP2D6:NM 001025161.2:exon1	p.P34S (c.C100T)
CYP3A5	.	.
ERCC1	ERCC1:NM 202001.2:exon3	p.N118N (c.T354C)
GSTT1	.	.
MTHFR	MTHFR:NM 005957.4:exon5	p.A222V (c.C665T)
UGT1A1	UGT1A1:NM 000463.2	.
XRCC1	XRCC1:NM 006297.2:exon10	p.Q399R (c.A1196G)
CDKN2A	CDKN2A:NM 000077.4:exon2	p.G67V (c.G200T)
EGFR	EGFR:NM 005228.3:exon19	p.745 750del (c.2235 2249delGGAAT
HGF	HGF:NM 000601.4:exon6	p.E210D (c.A630C)
TP53	TP53:NM 001126112.2:exon7	p.R248Q (c.G743A)
EGFR	.	.
NRAS	.	.

ExonicFunc	AF
missense variant	.
missense variant	.
missense variant	.
intron variant	.
synonymous variant	.
.	.
missense variant	.
upstream gene variant	.
missense variant	.
missense variant	99.6%
inframe deletion	56.4%
missense variant	24.1%
missense variant	99.7%
.	.
.	.
